# Crystal structure and Hirshfeld surface analysis of phenyl­alanine hydro­bromide

**DOI:** 10.1107/S2056989026010406

**Published:** 2026-10-20

**Authors:** Olesia I. Kucheriv, Alexandra Bargan, Vadim Potaskalov, Oleksii Brusylovets, Olena Prysiazhna

**Affiliations:** a Department of Chemistry, Taras Shevchenko National University of Kyiv, Volodymyrska St. 64, Kyiv 01601, Ukraine; b Department of Inorganic Polymers, Petru Poni Institute of Macromolecular Chemistry, Aleea Grigore Ghica Voda 41-A, Iasi 700487, Romania; c Department of General and Inorganic Chemistry, National Technical University of Ukraine Igor Sikorsky Kyiv Polytechnic Institute, Beresteiskyi Pr. 37, Kyiv 03056, Ukraine; d Bakul Institute for Superhard Materials, National Academy of Sciences of Ukraine, Avtozavodska St. 2, Kyiv 04074, Ukraine; e Institute of Innovative Education of Kyiv National University of Construction and Architecture, 4 Osvity Street, Kyiv 03037, Ukraine; Institute of Chemistry, Chinese Academy of Sciences

**Keywords:** crystal structure, amino acid salt, phenyl­alanine, bromide

## Abstract

Amino acid salts represent a diverse class of mol­ecular solids in which ionic inter­actions and hy­dro­gen bonding between protonated amino acid species and counter-ions govern the formation of supra­molecular architectures. Phenyl­alanine hydro­bromide (systematic name: 1-carb­oxy-2-phenyl­ethan-1-aminium bromide, C_9_H_12_NO_2_^+^·Br^−^) crystallizes in the chiral ortho­rhom­bic space group *P*2_1_2_1_2_1_. Its structure is built from protonated phenyl­alaninium cations, which inter­act with bromide anions through a network of N—H⋯Br and O—H⋯Br hy­dro­gen bonds, forming bilayers propagating parallel to the *ab* plane, with the polar groups oriented toward the inter­ior of the bilayers. The crystal structure is additionally consolidated by three types of weak T-shaped C(*sp*^2^)—H⋯π inter­actions.

## Abstract 2

The crystal structure of phenyl­alanine hydro­bromide reveals a hy­dro­gen-bonded bilayer architecture further consolidated by weak C(*sp*^2^)—H⋯π inter­actions.

## 1. Chemical context

Amino acids are fundamental building blocks of living systems and play a central role in biological processes, while their diverse chemical functionality also makes them important mol­ecular com­ponents in supra­molecular and materials chemistry (Wang *et al.*, 2024 ▸). In the solid state, amino acids commonly exist as zwitterions and form extended networks through hy­dro­gen bonding and electrostatic inter­actions (Görbitz, 2015 ▸; Moggach *et al.*, 2015 ▸). Consequently, knowledge of their crystal structures is essential for understanding the relationship between mol­ecular structure, inter­molecular inter­actions and the resulting properties of the solid phase. Amino acids have therefore long attracted attention in crystallographic studies, providing important model systems for investigating hy­dro­gen-bonding patterns, mol­ecular packing, polymorphism and structure–property relationships (Yamada *et al.*, 2008 ▸; de Moraes *et al.*, 2018 ▸). Modification of the ionic environment through protonation of the amino group and incorporation of inorganic counter-ions offers an additional means of controlling crystal packing and generating new structural motifs (Fleck & Petrosyan, 2014 ▸). In particular, halide salts of amino acids provide useful model systems in which the hy­dro­gen-bonding functionality of the organic com­ponent is combined with different inorganic counter-ions. Their crystal structures offer insight into how the nature of the halide and the presence of solvent mol­ecules influence inter­molecular inter­actions, crystal packing and the resulting supra­molecular organization (Petrosyan *et al.*, 2020 ▸).

Amino acid halide salts have also emerged as useful precursors and building blocks for hybrid organic–inorganic perovskites. Hybrid halide perovskites have attracted considerable attention due to their com­positional and structural tunability, which gives rise to a broad range of optical, electronic and other functional properties (Kucheriv *et al.*, 2023 ▸; Kucheriv *et al.*, 2025 ▸). The incorporation of amino-acid-derived cations into metal–halide frameworks enables the introduction of chirality and additional hy­dro­gen-bonding inter­actions, while retaining the characteristic structural flexibility of hybrid halide materials. Sirenko and co-workers demonstrated that α-alanine can serve as an organic com­ponent of chiral lead–halide hybrid perovskites, yielding (AlaH)Pb*X*_3_ com­pounds with distinct low-dimensional inorganic architectures and semiconducting optical properties (Sirenko *et al.*, 2021 ▸). Similar structural principles were subsequently extended to l-histidine, affording two-dimensional hybrid perovskites in which the amino acid cations separate the inorganic layers and induce noncentrosymmetric structures (Sirenko *et al.*, 2022 ▸). More recently, proline-based lead bromide perovskites further demonstrated the substantial structural diversity accessible through the use of amino-acid-derived organic cations (Sirenko *et al.*, 2023 ▸). These examples highlight the importance of understanding the crystal structures and inter­molecular inter­actions of amino acid halide salts themselves, as such structural information is relevant to their role as mol­ecular building blocks for the rational development of functional hybrid materials.

In this article, we report the crystal structure of phenyl­alaninium bromide and discuss its structural features and inter­molecular inter­actions.
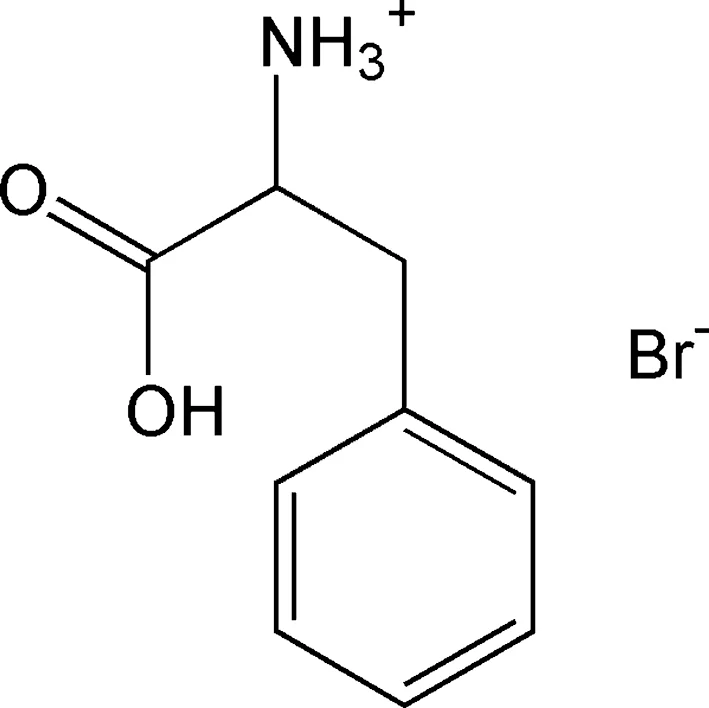


## 2. Structural commentary

The title salt crystallizes in the space group *P*2_1_2_1_2_1_. The asymmetric unit contains three protonated phenyl­alaninium cations and three bromide anions (Fig. 1 ▸). All bond lengths and angles are within the expected range. The phenylalaninium cation containing the C1–C6 aromatic ring is denoted as **A**, C10–C15 as **B** and C19–C24 as **C**. The C1—C7—C8—N1 and C10—C16—C17—N2 torsion angles in **A** and **B** are 56.6 (3) and 57.4 (3)°, respectively, indicating that these cations adopt anti­clinal conformation. The C19—C25—C26—N3 torsion angle in **C** is 168.8 (2)°, which is close to an anti­periplanar conformation. Due to such conformations, the **A** and **B** cations feature inter­molecular C2—H2⋯O1 and C15—H15⋯O3 hy­dro­gen bonds, while such an inter­action is geometrically impossible in **C**. The detailed geometric parameters of the hy­dro­gen bonds which are formed in this crystal structure are given in Table 1 ▸. The carb­oxy­lic acid group exhibits the expected asymmetric C—O bond lengths in all three types of phenyl­alaninium cations, with the C=O and C—OH bonds measuring *ca* 1.20 and 1.35 Å, respectively, in all three cases. This pronounced differentiation is consistent with the presence of a localized carbonyl and hydroxyl functionality rather than a delocalized carboxyl­ate group.

## 3. Supra­molecular features

Phenyl­alaninium cations form an extensive network of N—H⋯Br and O—H⋯Br hy­dro­gen bonds with bromide counter-ions (Table 1 ▸). Phenyl­alaninium cations are arranged in double layers propagating along the *ab* plane. Additional connection inside the layer is ensured by weak C8—H8⋯O2^iv^ and C17—H17⋯O4^v^ (Table 1 ▸) contacts. The carboxyl groups and ammonium cations face inside the double layers (Fig. 2 ▸). These double layers inter­act with each other by weak T-shaped C(*sp*^2^)—H⋯π inter­actions. In total, three types of C(*sp*^2^)—H⋯π inter­actions are formed: C24—H24⋯π(**C**) of 2.2898 (12) Å, C3—H3⋯π(**B**) of 2.6528 (12) Å and C14—H14⋯π(**A**) of 2.7747 (11) Å (Fig. 3 ▸).

## 4. Hirshfeld surface analysis

Hirshfeld surface analysis was performed separately for each crystallographically independent phenyl­alaninium cation. The Hirshfeld surfaces mapped over *d*_norm_ were generated at the standard resolution to visualize and characterize the inter­molecular contacts in the crystal structure [Figs. 4 ▸(*a*)–(*f*)]. A fixed colour scale was used, with white regions corresponding to contacts close to the sum of the van der Waals radii, whereas shorter and longer contacts are indicated by red and blue regions, respectively. In all three cases, the most pronounced red regions correspond to the shortest N—H⋯Br and N—H⋯O contacts. The associated two-dimensional fingerprint plots provide a qu­anti­tative representation of the inter­molecular contacts and their contributions to the crystal packing.

For all three cations, the most significant weak inter­molecular contacts are O⋯H/H⋯O, C⋯H/H⋯C and Br⋯H/H⋯Br, each contributing approximately 15–20% to the Hirshfeld surface. The exact contributions vary slightly among the three crystallographically independent cations and are given in Figs. 4 ▸(*b*)–(*d*). The O⋯H/H⋯O contacts arise from both C—H⋯O and N—H⋯O inter­actions, whereas the Br⋯H/H⋯Br contacts are primarily associated with hy­dro­gen bonding between the protonated ammonium or hydroxyl groups and bromide anions. The C⋯H/H⋯C contacts are associated with C—H⋯π inter­actions present in the crystal structure.

The remaining contribution to the fingerprint plots arises predominantly from H⋯H contacts. Although these contacts contribute appreciably to the overall Hirshfeld surface, they do not correspond to specific directional inter­molecular inter­actions and mainly originate from H atoms at the terminal positions of the mol­ecular fragments.

## 5. Database survey

A survey of the Cambridge Structural Database (CSD, Version 6.01, update of Fabruary 2026; Groom *et al.*, 2016 ▸) revealed one chemically related structure of l-phenyl­alanine l-phenyl­alaninium bromide (CADLOK01), which contained cocrystallized l-phenyl­alanine and l-phenyl­alaninium bromide in a 1:1 ratio (Surekha *et al.*, 2014 ▸).

## 6. Synthesis and crystallization

100 mg of phenyl­alanine were dissolved in 100 µl of con­cen­tra­ted (48%) HBr under heating and left to cool to room tem­per­a­ture. Within 1 h, colourless crystals suitable for X-ray analysis formed. The obtained crystals were stired in the mother liquor prior to measurement.

## 7. Refinement

Crystal data, data collection and structure refinement details are summarized in Table 2 ▸. H atoms were placed at calculated positions and refined isotropically with *U*_iso_(H) = 1.2*U*_eq_(C), 1.2*U*_eq_(N) or 1.5*U*_eq_(O). Ternary CH, secondary CH_2_ and aromatic H were refined with riding coordinates. Idealized NH_3_ and idealized tetra­hedral OH were refined as rotating groups with stretchable bonds.

## Supplementary Material

Crystal structure: contains datablock(s) I. DOI: 10.1107/S2056989026010406/nx2040sup1.cif

Structure factors: contains datablock(s) I. DOI: 10.1107/S2056989026010406/nx2040Isup2.hkl

CCDC reference: 2593846

Additional supporting information:  crystallographic information; 3D view; checkCIF report

## Figures and Tables

**Figure 1:**
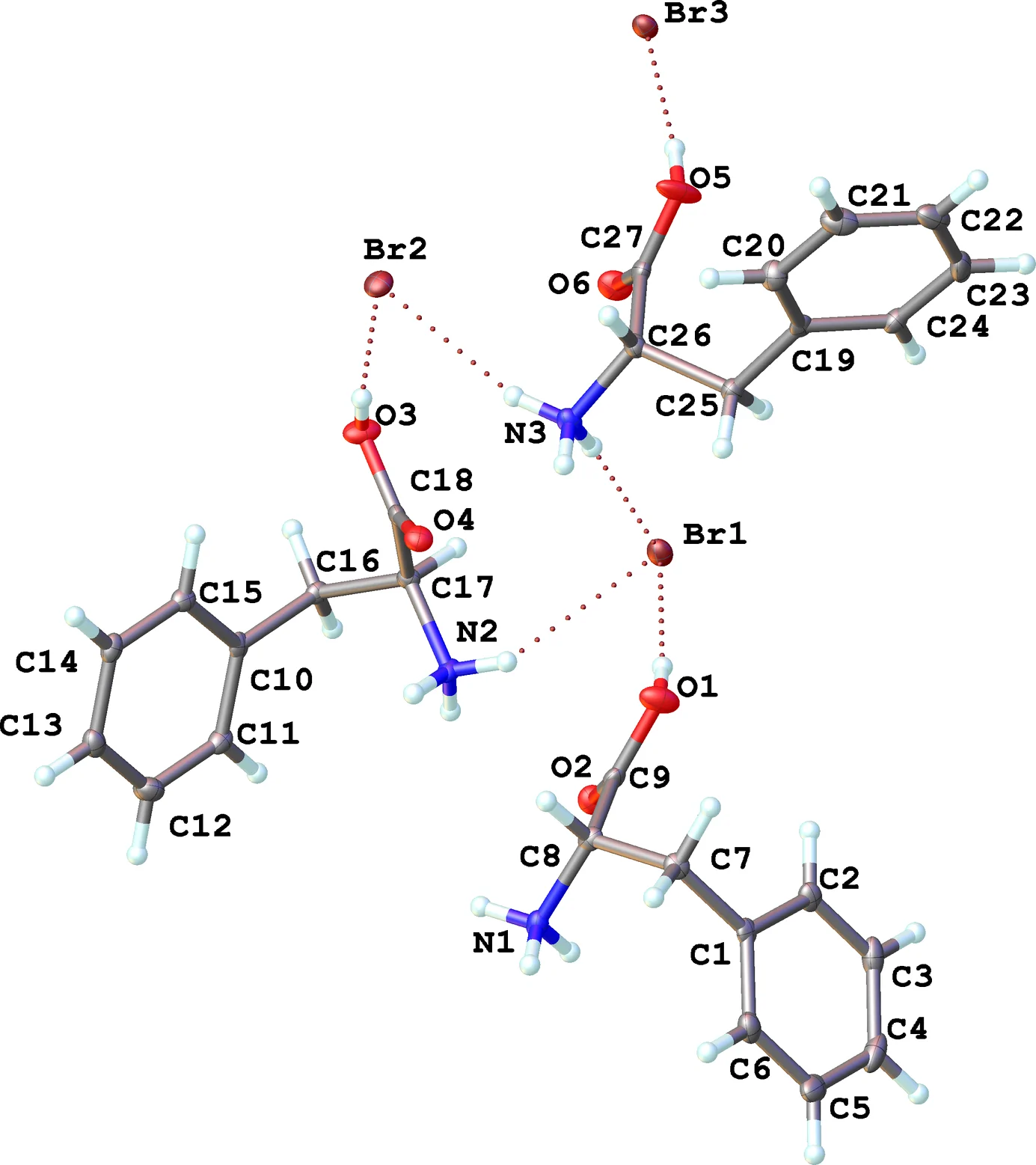
The asymmetric unit of the title com­pound, showing the atom-labelling scheme. Displacement ellipsoids are drawn at the 50% probability level.

**Figure 2:**
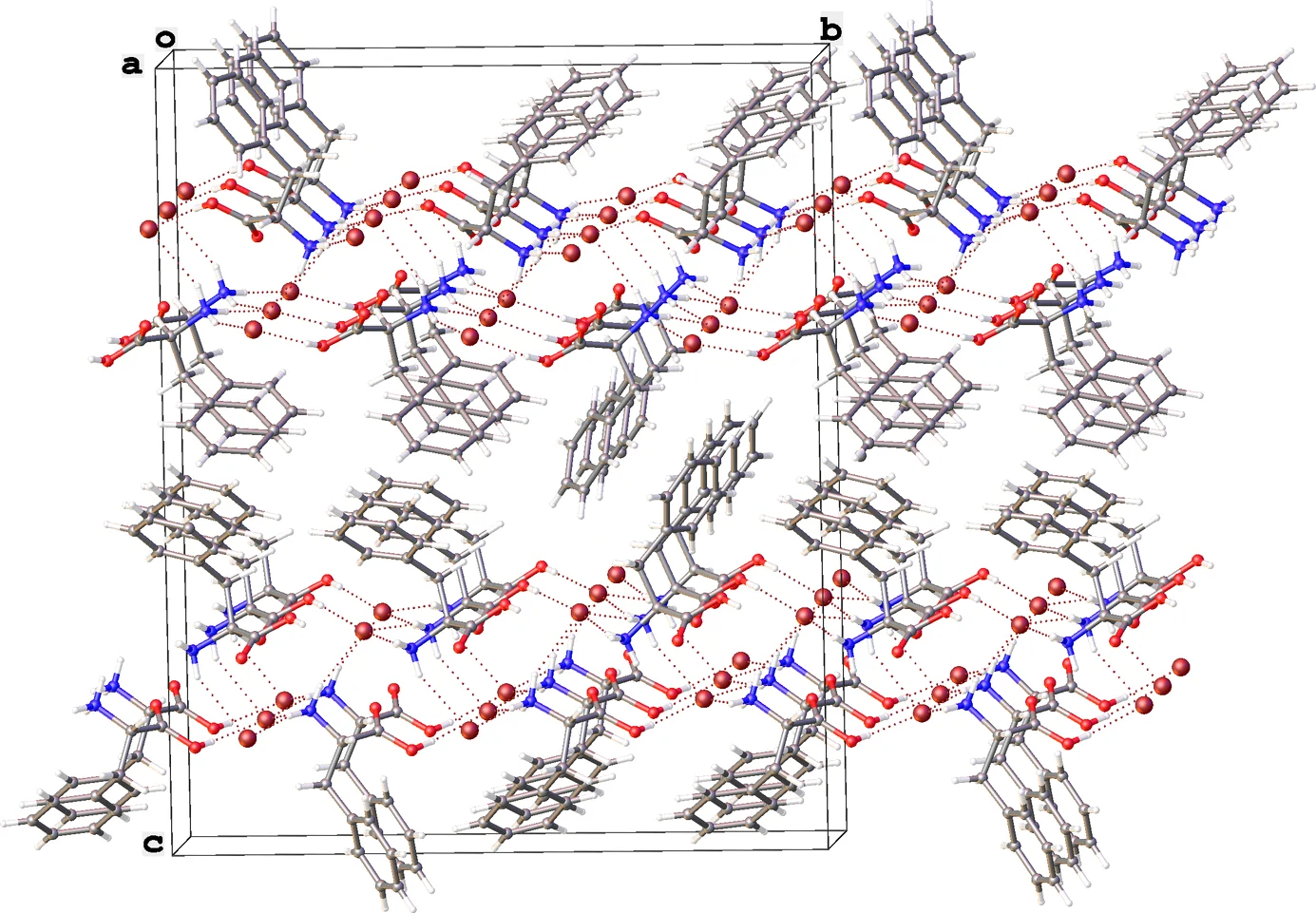
Fragment of the title com­pound, showing propagation of the supra­molecular bilayers in the *ab* plane.

**Figure 3:**
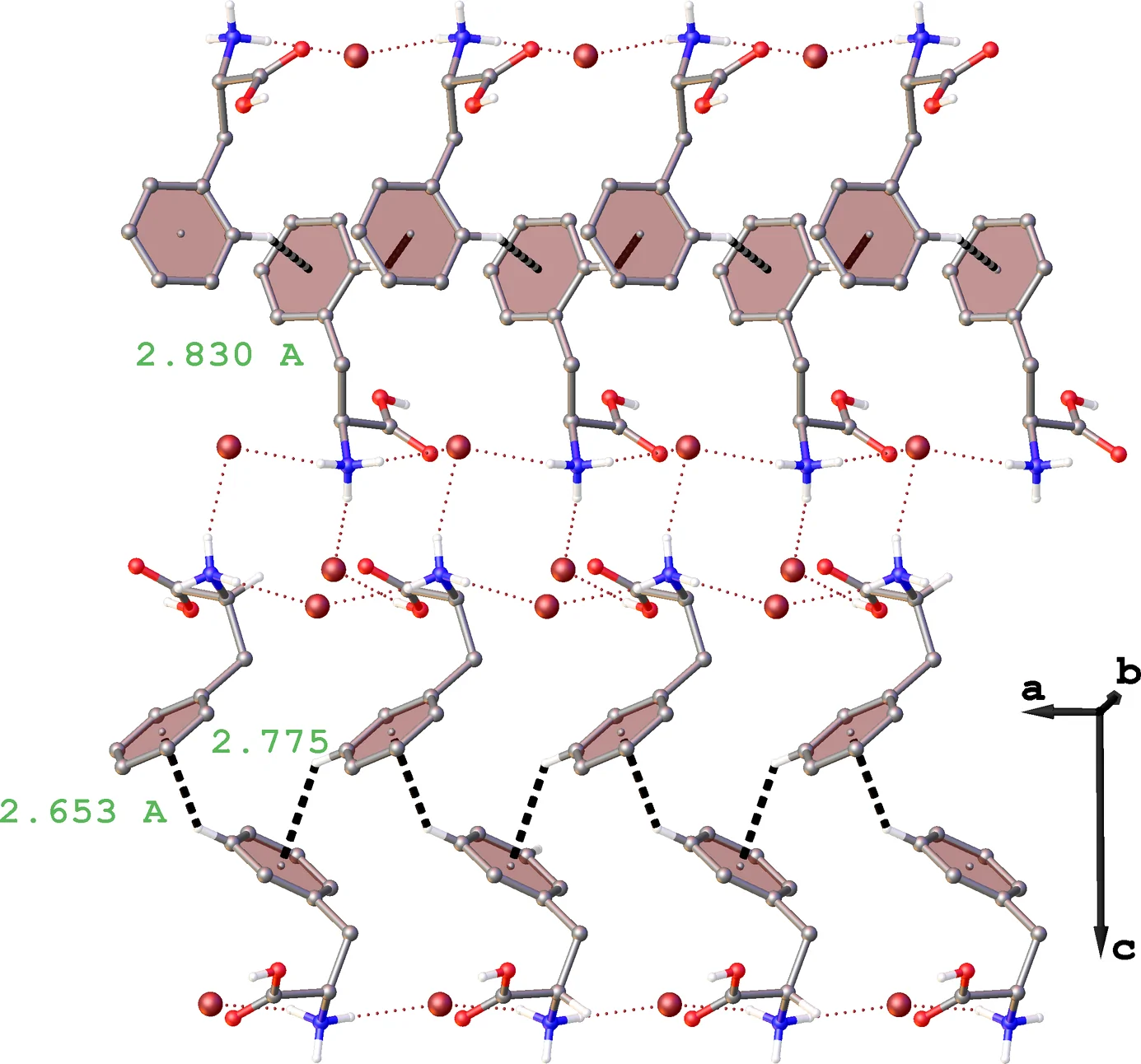
Fragment of the title com­pound showing C(*sp*^2^)—H⋯π inter­actions.

**Figure 4:**
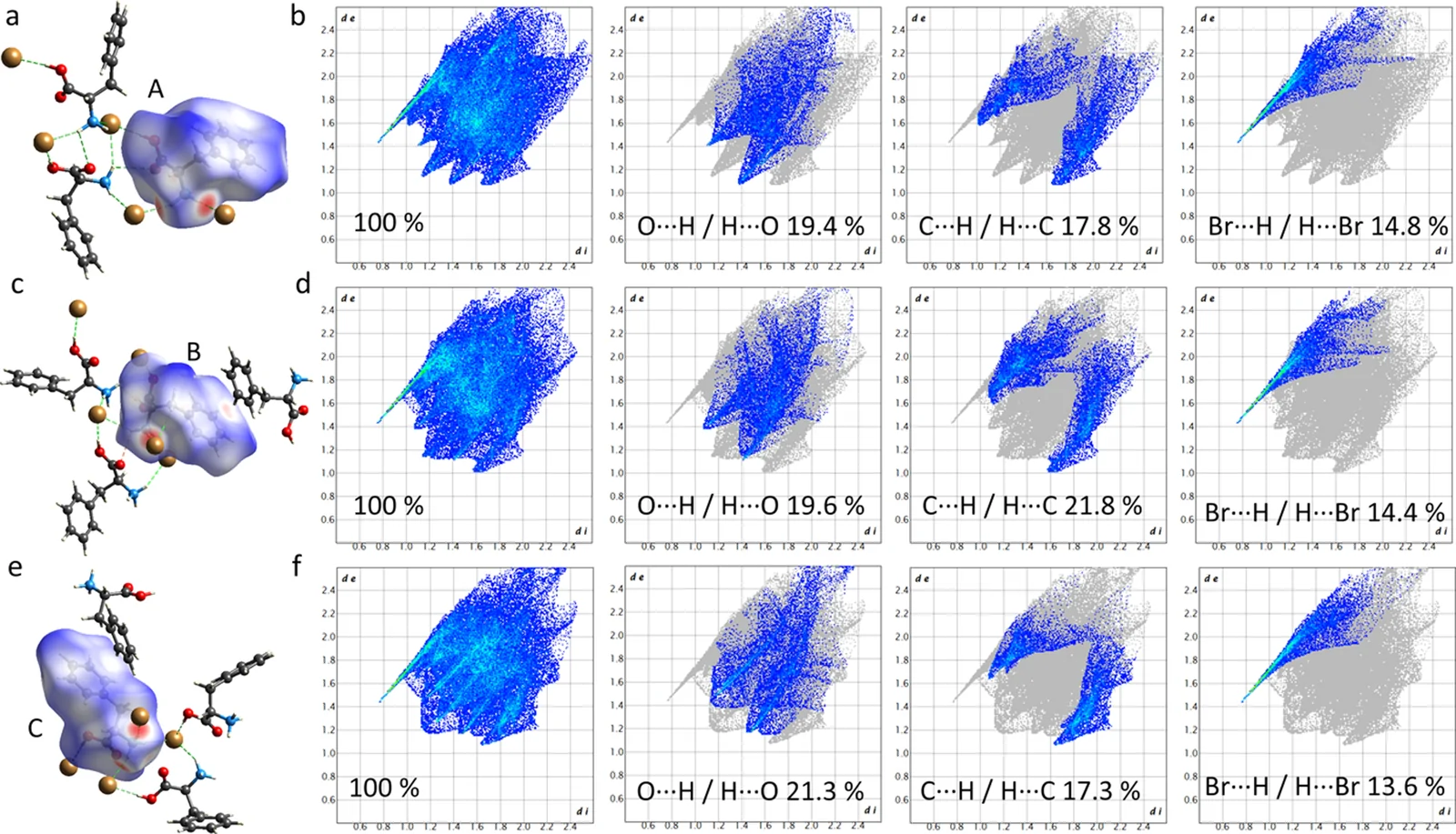
Hirshfeld surface and fingerprint plots of the (*a*)/(*b*) **A**, (*c*)/(*d*) **B** and (*e*)/(*f*) **C** phenyl­alaninium cations.

**Table 1:** Hydrogen-bond geometry (Å, °)

*D*—H⋯*A*	*D*—H	H⋯*A*	*D*⋯*A*	*D*—H⋯*A*
O1—H1⋯Br1	0.75	2.43	3.175 (2)	175
O3—H3*A*⋯Br2	0.76	2.41	3.165 (2)	173
O5—H5*A*⋯Br3	0.85	2.34	3.183 (2)	173
N1—H1*A*⋯Br2^i^	0.89	2.56	3.408 (2)	160
N1—H1*B*⋯Br3^i^	0.89	2.48	3.293 (2)	154
N1—H1*B*⋯O6^i^	0.89	2.67	3.140 (3)	114
N1—H1*C*⋯Br2^ii^	0.89	2.45	3.332 (2)	171
N2—H2*A*⋯Br1	0.87	2.65	3.428 (2)	149
N2—H2*A*⋯O2	0.87	2.52	3.038 (3)	119
N2—H2*B*⋯Br3^iii^	0.87	2.44	3.301 (2)	170
N2—H2*C*⋯Br3^i^	0.87	2.54	3.391 (2)	164
N3—H3*B*⋯Br1	0.87	2.55	3.406 (2)	168
N3—H3*C*⋯Br2	0.87	2.47	3.309 (2)	162
N3—H3*C*⋯O4	0.87	2.59	2.913 (3)	103
N3—H3*D*⋯Br1^iv^	0.87	2.48	3.324 (2)	163
C2—H2⋯O1	0.95	2.81	3.487 (4)	129
C8—H8⋯O2^iv^	1.00	2.58	3.406 (3)	140
C12—H12⋯O5^i^	0.95	2.96	3.347 (4)	106
C15—H15⋯O3	0.95	2.77	3.449 (4)	129
C17—H17⋯O4^v^	1.00	2.60	3.460 (3)	144

Symmetry codes: (i) 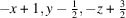; (ii) 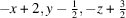; (iii) 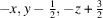; (iv) 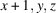; (v) 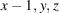.

**Table 2:** Experimental details

Crystal data	
Chemical formula	C_9_H_12_NO_2_^+^·Br^−^
*M*_r_	246.11
Crystal system, space group	Orthorhombic, *P*2_1_2_1_2_1_
Temperature (K)	100
*a*, *b*, *c* (Å)	5.5716 (1), 21.6685 (4), 25.9870 (5)
*V* (Å^3^)	3137.36 (10)
*Z*	12
Radiation type	Mo *K*α
μ (mm^−1^)	3.90
Crystal size (mm)	0.25 × 0.09 × 0.04

Data collection	
Diffractometer	Rigaku XtaLAB Synergy Dualflex diffractometer with a HyPix detector
Absorption correction	Analytical (*CrysAlis PRO*; Rigaku OD, 2024 ▸)
*T*_min_, *T*_max_	0.605, 0.880
No. of measured, independent and observed [*I* > 2σ(*I*)] reflections	20376, 7687, 6897
*R*_int_	0.027
(sin θ/λ)_max_ (Å^−1^)	0.709

Refinement	
*R*[*F*^2^ > 2σ(*F*^2^)], *wR*(*F*^2^), *S*	0.026, 0.046, 1.02
No. of reflections	7687
No. of parameters	364
H-atom treatment	H atoms treated by a mixture of independent and constrained refinement
Δρ_max_, Δρ_min_ (e Å^−3^)	0.39, −0.28
Absolute structure	Flack *x* determined using 2670 quotients [(*I*^+^)−(*I*^−^)]/[(*I*^+^)+ (*I*^−^)] (Parsons *et al.*, 2013 ▸)
Absolute structure parameter	−0.008 (4)

Computer programs: *CrysAlis PRO* (Rigaku OD, 2024 ▸), *SHELXT2018* (Sheldrick, 2015*a* ▸), *SHELXL2018* (Sheldrick, 2015*b* ▸) and *OLEX2* (Dolomanov *et al.*, 2009 ▸).

## supplementary crystallographic information

### 1-Carboxy-2-phenylethan-1-aminium bromide Crystal data

**Table:**

C_9_H_12_NO_2_^+^·Br^−^	*D*_x_ = 1.563 Mg m^−^^3^
*M**_r_* = 246.11	Mo *K*α radiation, λ = 0.71073 Å
Orthorhombic, *P*2_1_2_1_2_1_	Cell parameters from 11408 reflections
*a* = 5.5716 (1) Å	θ = 2.5–29.5°
*b* = 21.6685 (4) Å	µ = 3.90 mm^−^^1^
*c* = 25.9870 (5) Å	*T* = 100 K
*V* = 3137.36 (10) Å^3^	Block, colourless
*Z* = 12	0.25 × 0.09 × 0.04 mm
*F*(000) = 1488	

### 1-Carboxy-2-phenylethan-1-aminium bromide Data collection

**Table:**

Rigaku XtaLAB Synergy Dualflex diffractometer with a HyPix detector	7687 independent reflections
Radiation source: micro-focus sealed X-ray tube, PhotonJet (Mo) X-ray Source	6897 reflections with *I* > 2σ(*I*)
Mirror monochromator	*R*_int_ = 0.027
Detector resolution: 10.0000 pixels mm^-1^	θ_max_ = 30.2°, θ_min_ = 2.5°
ω scans	*h* = −6→7
Absorption correction: analytical (CrysAlis PRO; Rigaku OD, 2024)	*k* = −24→29
*T*_min_ = 0.605, *T*_max_ = 0.880	*l* = −32→29
20376 measured reflections	

### 1-Carboxy-2-phenylethan-1-aminium bromide Refinement

**Table:**

Refinement on *F*^2^	Hydrogen site location: inferred from neighbouring sites
Least-squares matrix: full	H atoms treated by a mixture of independent and constrained refinement
*R*[*F*^2^ > 2σ(*F*^2^)] = 0.026	*w* = 1/[σ^2^(*F*_o_^2^) + (0.0197*P*)^2^] where *P* = (*F*_o_^2^ + 2*F*_c_^2^)/3
*wR*(*F*^2^) = 0.046	(Δ/σ)_max_ = 0.002
*S* = 1.02	Δρ_max_ = 0.39 e Å^−^^3^
7687 reflections	Δρ_min_ = −0.28 e Å^−^^3^
364 parameters	Absolute structure: Flack *x* determined using 2670 quotients [(I+)-(I-)]/[(I+)+(I-)] (Parsons *et al.*, 2013)
0 restraints	Absolute structure parameter: −0.008 (4)

### 1-Carboxy-2-phenylethan-1-aminium bromide Special details

**Table:**

Geometry. All esds (except the esd in the dihedral angle between two l.s. planes) are estimated using the full covariance matrix. The cell esds are taken into account individually in the estimation of esds in distances, angles and torsion angles; correlations between esds in cell parameters are only used when they are defined by crystal symmetry. An approximate (isotropic) treatment of cell esds is used for estimating esds involving l.s. planes.

### 1-Carboxy-2-phenylethan-1-aminium bromide Fractional atomic coordinates and isotropic or equivalent isotropic displacement parameters (Å^2^)

**Table:**

	*x*	*y*	*z*	*U*_iso_*/*U*_eq_	
Br1	0.17873 (4)	0.65257 (2)	0.67621 (2)	0.01527 (7)	
Br2	0.78091 (5)	0.82203 (2)	0.80185 (2)	0.01596 (7)	
Br3	0.25455 (4)	0.99336 (2)	0.68558 (2)	0.01323 (7)	
O1	0.5903 (4)	0.55574 (10)	0.65121 (9)	0.0224 (5)	
H1	0.489 (6)	0.5774 (13)	0.6558 (11)	0.034*	
O2	0.3724 (3)	0.49088 (9)	0.70016 (9)	0.0161 (5)	
O3	0.3734 (4)	0.72749 (9)	0.83319 (9)	0.0201 (5)	
H3A	0.479 (6)	0.7486 (12)	0.8276 (12)	0.030*	
O4	0.5867 (3)	0.65787 (10)	0.78801 (8)	0.0148 (4)	
O5	0.5947 (4)	0.88562 (10)	0.64538 (9)	0.0275 (6)	
H5A	0.496 (6)	0.9136 (13)	0.6539 (12)	0.041*	
O6	0.3590 (3)	0.82479 (10)	0.69374 (8)	0.0196 (5)	
N1	0.7272 (4)	0.40849 (10)	0.70936 (9)	0.0134 (5)	
H1A	0.592 (3)	0.3912 (6)	0.6987 (5)	0.016*	
H1B	0.712 (3)	0.4195 (2)	0.7421 (6)	0.016*	
H1C	0.847 (3)	0.3817 (6)	0.7063 (6)	0.016*	
N2	0.2357 (4)	0.57364 (10)	0.78988 (9)	0.0125 (5)	
H2A	0.250 (3)	0.58000 (18)	0.7569 (6)	0.015*	
H2B	0.118 (2)	0.5481 (5)	0.7955 (6)	0.015*	
H2C	0.369 (3)	0.5579 (5)	0.8018 (5)	0.015*	
N3	0.6877 (4)	0.73528 (10)	0.69898 (9)	0.0140 (5)	
H3B	0.546 (3)	0.7193 (6)	0.6942 (4)	0.017*	
H3C	0.696 (3)	0.7508 (3)	0.7298 (5)	0.017*	
H3D	0.796 (3)	0.7067 (6)	0.6954 (5)	0.017*	
C1	0.7015 (5)	0.40577 (13)	0.59356 (10)	0.0124 (6)	
C2	0.4939 (5)	0.42966 (14)	0.57039 (12)	0.0149 (6)	
H2	0.459171	0.472434	0.573325	0.018*	
C3	0.3396 (5)	0.39180 (16)	0.54340 (12)	0.0200 (7)	
H3	0.198754	0.408471	0.528201	0.024*	
C4	0.3900 (6)	0.32964 (16)	0.53849 (12)	0.0237 (8)	
H4	0.284106	0.303673	0.519730	0.028*	
C5	0.5940 (6)	0.30515 (15)	0.56078 (12)	0.0211 (7)	
H5	0.628197	0.262391	0.557441	0.025*	
C6	0.7497 (5)	0.34346 (14)	0.58824 (10)	0.0174 (6)	
H6	0.890139	0.326542	0.603431	0.021*	
C7	0.8698 (4)	0.44712 (14)	0.62374 (11)	0.0130 (6)	
H7A	0.896221	0.485554	0.603939	0.016*	
H7B	1.026675	0.426083	0.627167	0.016*	
C8	0.7793 (4)	0.46417 (12)	0.67745 (11)	0.0124 (6)	
H8	0.909917	0.487770	0.695034	0.015*	
C9	0.5549 (5)	0.50447 (13)	0.67785 (12)	0.0129 (6)	
C10	0.2838 (5)	0.58765 (13)	0.90514 (10)	0.0113 (6)	
C11	0.2425 (5)	0.52576 (13)	0.91687 (11)	0.0140 (6)	
H11	0.103093	0.505814	0.903990	0.017*	
C12	0.4030 (5)	0.49308 (15)	0.94713 (12)	0.0181 (7)	
H12	0.374020	0.450834	0.954657	0.022*	
C13	0.6066 (5)	0.52212 (14)	0.96649 (12)	0.0156 (7)	
H13	0.715685	0.499804	0.987537	0.019*	
C14	0.6502 (5)	0.58351 (14)	0.95512 (11)	0.0139 (6)	
H14	0.790007	0.603240	0.968066	0.017*	
C15	0.4894 (4)	0.61617 (14)	0.92475 (11)	0.0127 (6)	
H15	0.519442	0.658375	0.917216	0.015*	
C16	0.1064 (4)	0.62332 (13)	0.87211 (11)	0.0109 (6)	
H16A	0.078216	0.664093	0.888195	0.013*	
H16B	−0.048354	0.600878	0.871971	0.013*	
C17	0.1859 (4)	0.63338 (12)	0.81636 (11)	0.0113 (6)	
H17	0.051278	0.654129	0.797699	0.014*	
C18	0.4083 (5)	0.67351 (13)	0.81056 (11)	0.0120 (6)	
C19	0.8038 (5)	0.79904 (12)	0.56348 (10)	0.0120 (6)	
C20	1.0195 (5)	0.83066 (13)	0.56674 (12)	0.0178 (7)	
H20	1.112471	0.828004	0.597308	0.021*	
C21	1.1015 (5)	0.86625 (14)	0.52580 (13)	0.0218 (7)	
H21	1.249390	0.887782	0.528716	0.026*	
C22	0.9702 (6)	0.87050 (14)	0.48114 (13)	0.0219 (7)	
H22	1.026983	0.894670	0.453172	0.026*	
C23	0.7545 (5)	0.83918 (14)	0.47738 (11)	0.0213 (7)	
H23	0.662621	0.841877	0.446675	0.026*	
C24	0.6716 (5)	0.80379 (13)	0.51828 (11)	0.0143 (6)	
H24	0.522950	0.782619	0.515326	0.017*	
C25	0.7183 (5)	0.75706 (12)	0.60608 (10)	0.0131 (6)	
H25A	0.816393	0.718980	0.605500	0.016*	
H25B	0.550219	0.745061	0.598786	0.016*	
C26	0.7294 (5)	0.78472 (12)	0.66055 (10)	0.0125 (6)	
H26	0.891551	0.803235	0.666316	0.015*	
C27	0.5384 (5)	0.83380 (13)	0.66893 (11)	0.0138 (7)	

### 1-Carboxy-2-phenylethan-1-aminium bromide Atomic displacement parameters (Å^2^)

**Table:**

	*U*^11^	*U*^22^	*U*^33^	*U*^12^	*U*^13^	*U*^23^
Br1	0.01505 (13)	0.01604 (15)	0.01472 (16)	0.00555 (11)	−0.00005 (11)	0.00074 (13)
Br2	0.01382 (14)	0.01551 (14)	0.01854 (16)	−0.00551 (11)	−0.00003 (11)	−0.00128 (12)
Br3	0.01307 (12)	0.01245 (14)	0.01418 (15)	0.00420 (11)	0.00076 (12)	0.00126 (11)
O1	0.0219 (11)	0.0155 (12)	0.0299 (15)	0.0056 (9)	0.0108 (10)	0.0035 (11)
O2	0.0123 (9)	0.0187 (12)	0.0172 (12)	0.0027 (8)	0.0036 (8)	−0.0003 (9)
O3	0.0194 (11)	0.0140 (11)	0.0268 (14)	−0.0051 (9)	0.0091 (9)	−0.0021 (10)
O4	0.0126 (9)	0.0149 (11)	0.0171 (12)	−0.0021 (9)	0.0022 (8)	0.0014 (10)
O5	0.0383 (13)	0.0114 (12)	0.0329 (16)	0.0103 (10)	0.0213 (11)	0.0066 (11)
O6	0.0156 (10)	0.0192 (11)	0.0241 (13)	0.0053 (8)	0.0052 (9)	0.0011 (10)
N1	0.0112 (11)	0.0174 (13)	0.0115 (12)	0.0032 (10)	−0.0006 (10)	−0.0016 (10)
N2	0.0131 (11)	0.0131 (12)	0.0111 (12)	−0.0053 (10)	0.0013 (10)	−0.0013 (9)
N3	0.0136 (11)	0.0148 (13)	0.0135 (13)	0.0045 (9)	0.0016 (10)	0.0010 (10)
C1	0.0135 (13)	0.0168 (15)	0.0067 (15)	0.0003 (11)	0.0036 (11)	0.0008 (11)
C2	0.0150 (14)	0.0187 (17)	0.0112 (17)	0.0050 (12)	0.0022 (11)	−0.0007 (13)
C3	0.0140 (14)	0.036 (2)	0.0105 (16)	0.0016 (14)	−0.0001 (12)	0.0012 (14)
C4	0.0240 (17)	0.035 (2)	0.0118 (18)	−0.0132 (14)	0.0011 (13)	−0.0045 (15)
C5	0.0331 (18)	0.0180 (17)	0.0121 (18)	−0.0059 (14)	0.0038 (13)	0.0013 (14)
C6	0.0195 (14)	0.0214 (15)	0.0114 (15)	0.0040 (14)	0.0001 (12)	0.0012 (12)
C7	0.0094 (13)	0.0155 (16)	0.0139 (17)	0.0006 (11)	0.0028 (11)	0.0008 (13)
C8	0.0094 (12)	0.0134 (14)	0.0145 (15)	0.0008 (10)	−0.0012 (12)	−0.0021 (12)
C9	0.0157 (13)	0.0115 (16)	0.0114 (17)	0.0024 (11)	−0.0007 (12)	−0.0044 (14)
C10	0.0110 (13)	0.0166 (15)	0.0063 (14)	0.0013 (11)	0.0028 (11)	−0.0010 (11)
C11	0.0143 (13)	0.0156 (14)	0.0121 (14)	−0.0040 (12)	0.0031 (12)	−0.0019 (11)
C12	0.0211 (15)	0.0135 (16)	0.020 (2)	0.0023 (13)	0.0024 (12)	0.0005 (14)
C13	0.0166 (14)	0.0188 (17)	0.0114 (18)	0.0039 (12)	0.0007 (12)	0.0004 (13)
C14	0.0112 (13)	0.0201 (16)	0.0103 (16)	−0.0010 (12)	0.0012 (11)	−0.0025 (12)
C15	0.0137 (13)	0.0128 (15)	0.0115 (17)	−0.0017 (11)	0.0038 (11)	−0.0004 (13)
C16	0.0104 (12)	0.0117 (16)	0.0107 (16)	−0.0003 (11)	0.0024 (11)	−0.0019 (12)
C17	0.0114 (12)	0.0102 (14)	0.0122 (15)	0.0011 (10)	0.0005 (11)	0.0005 (11)
C18	0.0151 (13)	0.0120 (15)	0.0089 (16)	−0.0016 (11)	−0.0038 (11)	0.0032 (13)
C19	0.0154 (14)	0.0111 (13)	0.0096 (14)	0.0043 (11)	0.0025 (11)	−0.0031 (12)
C20	0.0147 (14)	0.0212 (19)	0.0176 (17)	0.0013 (11)	−0.0010 (12)	0.0000 (14)
C21	0.0187 (15)	0.0173 (17)	0.029 (2)	0.0002 (12)	0.0082 (13)	0.0023 (15)
C22	0.0316 (17)	0.0144 (18)	0.020 (2)	0.0043 (13)	0.0104 (14)	0.0030 (14)
C23	0.0325 (16)	0.0213 (17)	0.0100 (15)	0.0064 (15)	−0.0024 (13)	−0.0018 (12)
C24	0.0152 (14)	0.0143 (15)	0.0133 (16)	−0.0010 (11)	0.0000 (11)	−0.0039 (12)
C25	0.0152 (14)	0.0125 (14)	0.0116 (14)	0.0003 (12)	0.0003 (11)	−0.0012 (11)
C26	0.0137 (13)	0.0133 (14)	0.0106 (14)	−0.0002 (12)	0.0000 (11)	0.0013 (11)
C27	0.0174 (14)	0.0141 (18)	0.0100 (17)	−0.0002 (12)	−0.0038 (11)	−0.0022 (13)

### 1-Carboxy-2-phenylethan-1-aminium bromide Geometric parameters (Å, º)

**Table:**

O1—H1	0.75 (4)	C8—H8	1.0000
O1—C9	1.324 (4)	C8—C9	1.525 (4)
O2—C9	1.207 (3)	C10—C11	1.394 (4)
O3—H3A	0.76 (4)	C10—C15	1.398 (4)
O3—C18	1.324 (3)	C10—C16	1.520 (4)
O4—C18	1.203 (3)	C11—H11	0.9500
O5—H5A	0.85 (4)	C11—C12	1.385 (4)
O5—C27	1.317 (3)	C12—H12	0.9500
O6—C27	1.205 (3)	C12—C13	1.391 (4)
N1—H1A	0.887 (15)	C13—H13	0.9500
N1—H1B	0.887 (15)	C13—C14	1.384 (4)
N1—H1C	0.887 (15)	C14—H14	0.9500
N1—C8	1.492 (3)	C14—C15	1.388 (4)
N2—H2A	0.872 (14)	C15—H15	0.9500
N2—H2B	0.872 (15)	C16—H16A	0.9900
N2—H2C	0.872 (15)	C16—H16B	0.9900
N2—C17	1.492 (3)	C16—C17	1.531 (4)
N3—H3B	0.871 (15)	C17—H17	1.0000
N3—H3C	0.871 (14)	C17—C18	1.521 (4)
N3—H3D	0.871 (14)	C19—C20	1.386 (4)
N3—C26	1.483 (3)	C19—C24	1.390 (4)
C1—C2	1.403 (4)	C19—C25	1.510 (4)
C1—C6	1.384 (4)	C20—H20	0.9500
C1—C7	1.516 (4)	C20—C21	1.391 (4)
C2—H2	0.9500	C21—H21	0.9500
C2—C3	1.380 (4)	C21—C22	1.375 (5)
C3—H3	0.9500	C22—H22	0.9500
C3—C4	1.382 (5)	C22—C23	1.384 (4)
C4—H4	0.9500	C23—H23	0.9500
C4—C5	1.381 (5)	C23—C24	1.390 (4)
C5—H5	0.9500	C24—H24	0.9500
C5—C6	1.397 (4)	C25—H25A	0.9900
C6—H6	0.9500	C25—H25B	0.9900
C7—H7A	0.9900	C25—C26	1.538 (4)
C7—H7B	0.9900	C26—H26	1.0000
C7—C8	1.529 (4)	C26—C27	1.520 (4)
			
C9—O1—H1	109.5	C11—C12—H12	120.0
C18—O3—H3A	109.5	C11—C12—C13	120.0 (3)
C27—O5—H5A	109.5	C13—C12—H12	120.0
H1A—N1—H1B	109.5	C12—C13—H13	120.0
H1A—N1—H1C	109.5	C14—C13—C12	120.0 (3)
H1B—N1—H1C	109.5	C14—C13—H13	120.0
C8—N1—H1A	109.5	C13—C14—H14	120.1
C8—N1—H1B	109.5	C13—C14—C15	119.9 (3)
C8—N1—H1C	109.5	C15—C14—H14	120.1
H2A—N2—H2B	109.5	C10—C15—H15	119.6
H2A—N2—H2C	109.5	C14—C15—C10	120.7 (3)
H2B—N2—H2C	109.5	C14—C15—H15	119.6
C17—N2—H2A	109.5	C10—C16—H16A	108.6
C17—N2—H2B	109.5	C10—C16—H16B	108.6
C17—N2—H2C	109.5	C10—C16—C17	114.8 (2)
H3B—N3—H3C	109.5	H16A—C16—H16B	107.6
H3B—N3—H3D	109.5	C17—C16—H16A	108.6
H3C—N3—H3D	109.5	C17—C16—H16B	108.6
C26—N3—H3B	109.5	N2—C17—C16	111.5 (2)
C26—N3—H3C	109.5	N2—C17—H17	107.8
C26—N3—H3D	109.5	N2—C17—C18	107.4 (2)
C2—C1—C7	120.9 (3)	C16—C17—H17	107.8
C6—C1—C2	118.5 (3)	C18—C17—C16	114.3 (2)
C6—C1—C7	120.6 (2)	C18—C17—H17	107.8
C1—C2—H2	119.6	O3—C18—C17	110.0 (2)
C3—C2—C1	120.8 (3)	O4—C18—O3	125.9 (3)
C3—C2—H2	119.6	O4—C18—C17	124.1 (3)
C2—C3—H3	120.0	C20—C19—C24	118.3 (3)
C2—C3—C4	120.0 (3)	C20—C19—C25	121.8 (3)
C4—C3—H3	120.0	C24—C19—C25	119.8 (2)
C3—C4—H4	119.9	C19—C20—H20	119.6
C5—C4—C3	120.2 (3)	C19—C20—C21	120.8 (3)
C5—C4—H4	119.9	C21—C20—H20	119.6
C4—C5—H5	120.1	C20—C21—H21	119.7
C4—C5—C6	119.8 (3)	C22—C21—C20	120.5 (3)
C6—C5—H5	120.1	C22—C21—H21	119.7
C1—C6—C5	120.7 (3)	C21—C22—H22	120.4
C1—C6—H6	119.7	C21—C22—C23	119.3 (3)
C5—C6—H6	119.7	C23—C22—H22	120.4
C1—C7—H7A	108.7	C22—C23—H23	119.8
C1—C7—H7B	108.7	C22—C23—C24	120.4 (3)
C1—C7—C8	114.3 (2)	C24—C23—H23	119.8
H7A—C7—H7B	107.6	C19—C24—H24	119.6
C8—C7—H7A	108.7	C23—C24—C19	120.7 (3)
C8—C7—H7B	108.7	C23—C24—H24	119.6
N1—C8—C7	112.1 (2)	C19—C25—H25A	108.5
N1—C8—H8	107.5	C19—C25—H25B	108.5
N1—C8—C9	107.4 (2)	C19—C25—C26	115.3 (2)
C7—C8—H8	107.5	H25A—C25—H25B	107.5
C9—C8—C7	114.5 (2)	C26—C25—H25A	108.5
C9—C8—H8	107.5	C26—C25—H25B	108.5
O1—C9—C8	110.8 (2)	N3—C26—C25	109.4 (2)
O2—C9—O1	125.6 (3)	N3—C26—H26	109.3
O2—C9—C8	123.7 (3)	N3—C26—C27	107.4 (2)
C11—C10—C15	118.7 (3)	C25—C26—H26	109.3
C11—C10—C16	120.4 (2)	C27—C26—C25	112.1 (2)
C15—C10—C16	120.9 (2)	C27—C26—H26	109.3
C10—C11—H11	119.7	O5—C27—C26	111.3 (2)
C12—C11—C10	120.6 (3)	O6—C27—O5	125.8 (3)
C12—C11—H11	119.7	O6—C27—C26	122.9 (3)
			
N1—C8—C9—O1	178.4 (2)	C11—C10—C16—C17	−103.3 (3)
N1—C8—C9—O2	−0.8 (4)	C11—C12—C13—C14	−0.7 (5)
N2—C17—C18—O3	179.6 (2)	C12—C13—C14—C15	0.6 (4)
N2—C17—C18—O4	0.5 (4)	C13—C14—C15—C10	−0.4 (4)
N3—C26—C27—O5	164.8 (2)	C15—C10—C11—C12	−0.4 (4)
N3—C26—C27—O6	−16.0 (4)	C15—C10—C16—C17	77.4 (3)
C1—C2—C3—C4	−0.7 (5)	C16—C10—C11—C12	−179.7 (3)
C1—C7—C8—N1	56.6 (3)	C16—C10—C15—C14	179.7 (3)
C1—C7—C8—C9	−66.1 (3)	C16—C17—C18—O3	−56.1 (3)
C2—C1—C6—C5	−0.5 (4)	C16—C17—C18—O4	124.7 (3)
C2—C1—C7—C8	76.6 (3)	C19—C20—C21—C22	−0.3 (5)
C2—C3—C4—C5	0.4 (5)	C19—C25—C26—N3	−168.8 (2)
C3—C4—C5—C6	−0.1 (5)	C19—C25—C26—C27	72.2 (3)
C4—C5—C6—C1	0.2 (4)	C20—C19—C24—C23	0.3 (4)
C6—C1—C2—C3	0.7 (4)	C20—C19—C25—C26	48.5 (4)
C6—C1—C7—C8	−103.4 (3)	C20—C21—C22—C23	0.3 (5)
C7—C1—C2—C3	−179.3 (3)	C21—C22—C23—C24	0.0 (4)
C7—C1—C6—C5	179.5 (3)	C22—C23—C24—C19	−0.3 (4)
C7—C8—C9—O1	−56.4 (3)	C24—C19—C20—C21	0.0 (4)
C7—C8—C9—O2	124.3 (3)	C24—C19—C25—C26	−135.3 (3)
C10—C11—C12—C13	0.5 (4)	C25—C19—C20—C21	176.2 (3)
C10—C16—C17—N2	57.4 (3)	C25—C19—C24—C23	−176.0 (2)
C10—C16—C17—C18	−64.6 (3)	C25—C26—C27—O5	−75.1 (3)
C11—C10—C15—C14	0.3 (4)	C25—C26—C27—O6	104.2 (3)

### 1-Carboxy-2-phenylethan-1-aminium bromide Hydrogen-bond geometry (Å, º)

**Table:**

*D*—H···*A*	*D*—H	H···*A*	*D*···*A*	*D*—H···*A*
O1—H1···Br1	0.75	2.43	3.175 (2)	175
O3—H3*A*···Br2	0.76	2.41	3.165 (2)	173
O5—H5*A*···Br3	0.85	2.34	3.183 (2)	173
N1—H1*A*···Br2^i^	0.89	2.56	3.408 (2)	160
N1—H1*B*···Br3^i^	0.89	2.48	3.293 (2)	154
N1—H1*B*···O6^i^	0.89	2.67	3.140 (3)	114
N1—H1*C*···Br2^ii^	0.89	2.45	3.332 (2)	171
N2—H2*A*···Br1	0.87	2.65	3.428 (2)	149
N2—H2*A*···O2	0.87	2.52	3.038 (3)	119
N2—H2*B*···Br3^iii^	0.87	2.44	3.301 (2)	170
N2—H2*C*···Br3^i^	0.87	2.54	3.391 (2)	164
N3—H3*B*···Br1	0.87	2.55	3.406 (2)	168
N3—H3*C*···Br2	0.87	2.47	3.309 (2)	162
N3—H3*C*···O4	0.87	2.59	2.913 (3)	103
N3—H3*D*···Br1^iv^	0.87	2.48	3.324 (2)	163
C2—H2···O1	0.95	2.81	3.487 (4)	129
C8—H8···O2^iv^	1.00	2.58	3.406 (3)	140
C12—H12···O5^i^	0.95	2.96	3.347 (4)	106
C15—H15···O3	0.95	2.77	3.449 (4)	129
C17—H17···O4^v^	1.00	2.60	3.460 (3)	144

Symmetry codes: (i) −*x*+1, *y*−1/2, −*z*+3/2; (ii) −*x*+2, *y*−1/2, −*z*+3/2; (iii) −*x*, *y*−1/2, −*z*+3/2; (iv) *x*+1, *y*, *z*; (v) *x*−1, *y*, *z*.
